# Supplementary material for: Mode attraction, rejection and control in nonlinear multimode optics
Source: Nat Commun. 2023 Nov 24;14:7704. doi: 10.1038/s41467-023-42869-0 (PMC10673906; doi:10.1038/s41467-023-42869-0)
Supplement: Supplementary file 1 — Supplementary Information [file 41467_2023_42869_MOESM1_ESM.pdf]

SUPPLEMENTARY INFORMATION:

Mode attraction, rejection and control in nonlinear multimode optics.

Kunhao Ji[1], Ian Davidson[1], Jayanta Sahu[1], David. J. Richardson[1,2], Stefan Wabnitz[3], Massimiliano Guasoni[1 *]

1. Optoelectronics Research Centre, University of Southampton, Southampton SO17 1BJ, United Kingdom

2. Microsoft (Lumenisity Limited), Unit 7, The Quadrangle, Abbey Park Industrial Estate, Romsey, SO51 9DL, United Kingdom

3. Department of Information Engineering, Electronics and Telecommunications (DIET), Sapienza University of Rome, 00184 Rome, Italy

Notation used: Eq.(X) and Fig.(X) refers respectively to the equation and figure number X in the manuscript; whereas Eq.(SX), Fig.(SX), Table (SX) refers to the equation, figure or table number X in the supplementary information discussed here below.

Note 1. Theory of mode rejection and mode attraction

In this section we report the details of the mathematics leading from the coupled Schrödinger equations Eqs.(1) to the relation for mode rejection Eq.(3). As mentioned in the manuscript (Methods) we focus on the stationary problem and on the case $\gamma_{nn}=\gamma$ and $\gamma_{nm}=(1/2)\gamma$ $(n \neq m)$. Under these conditions Eqs.(1) can be rewritten as:

$$\partial_z f_n = i\gamma f_n \sum_{\mathrm{m}}(k|b_m|^2 + |f_m|^2) + ik\gamma b_n^* \sum_m b_m f_m$$
$$-\partial_z b_n = i\gamma b_n \sum_{\mathrm{m}}(k|f_m|^2 + |b_m|^2) + ik\gamma f_n^* \sum_m b_m f_m \tag{S1}$$

Where $k = \kappa/2$. Eqs.(S1) are completed with the boundary conditions that fix the input forward signal (FS) in z=0 and the input backward control beam (BCB) in z=L, namely $f_n(0)$ and $b_n(L)$. We now make the following change of variables:

$$f_n = P_f^{1/2}\, \hat{f}_n e^{i\gamma(P_f + kP_b)z}$$
$$b_n = P_b^{1/2}\, \hat{b}_n e^{-i\gamma(P_b + kP_f)z} \tag{S2}$$

Where $P_f = \sum_n |f_n|^2$ and $P_b = \sum_n |b_n|^2$ are the total forward and backward powers and are conserved (propagation losses are negligible). In the following, our focus is on the derivation of an equation for the correlation coefficient $D_R = (\sum_n f_n\, b_n)/Q$, with $Q = (P_f\, P_b)^{1/2}$ and where the subscript R of D_R stands for rejection (in contrast to the coefficient D_A used when dealing with attraction phenomena, see later). Note that, according to Eqs.(S2), $\sum_n |\hat{f}_n|^2 = \sum_n |\hat{b}_n|^2 = 1$. Moreover, D_R and $\widehat{D}_R = \sum_n \hat{f}_n \hat{b}_n$ are identical up to a phase term $e^{j\Delta P(k-1)z}$, with $\Delta P = P_b - P_f$, and consequently $|\widehat{D}_R| = |D_R|$. By inserting Eqs.(S2) into Eqs.(S1), we find the following relations:

$$\partial_z \hat{f}_n = i\gamma k P_b \widehat{D}_R \hat{b}_n^*$$
$$\partial_z \hat{b}_n^* = i\gamma k P_f \widehat{D}_R^* \hat{\mathrm{f}}_{\mathrm{n}} \tag{S3}$$

If we calculate $\partial_z \widehat{D}_R = \sum_n \partial_z(\hat{f}_n\, \hat{b}_n) = \sum_n[\partial_z(\hat{f}_n)\, \hat{b}_n + \partial_z(\hat{b}_n)\, \hat{f}_n]$ we find a simple equation that can be solved analytically:

$$\partial_z \widehat{D}_R = i\gamma k\, \widehat{D}_R\, \Delta P \tag{S4}$$

The solution of Eq.(S4) reads $\widehat{D}_R(z) = \widehat{D}_R(0)\, e^{i\,\gamma\, k\, \Delta P\, z}$. Note that at this point $\widehat{D}_R(z)$ *is* still undetermined. Indeed, due to the counter-propagating nature of the system, $\widehat{D}_R(0)$ is unknown since it

depends on $b_n(0)$. However, we can now insert the solution of Eq.(S4) into Eq.(S3) and solve the latter for $\hat{f}_n(L)$, which after some algebra brings to the following equality:

$$\hat{f}_n(L) = \frac{\hat{f}_n(0)\, h\, e^{\frac{1}{2}i\gamma\, k\, \Delta P\, L}\, i - \hat{b}_n^*(L)\, \hat{D}_R(L)\, \sin(Th)\, (v+w)}{h\, \cos(T\, h)\, i - v\, \sin(Th)} \tag{S5}$$

With $v = \Delta P/(2Q)$; $w = P_{tot}/(2Q)$; $h^2 = v^2 + \left|\hat{D}_R(L)\right|^2$; $T = k\gamma QL$; $Q^2 = P_f P_b$; $P_{tot} = P_f + P_b$. If now we multiply the left-hand-side and right-hand-side of Eq.(S5) by $\hat{b}_n(L)$ and we take the summation over the modes, we find:

$$\overbrace{\sum_n \hat{f}_n(L)\hat{b}_n(L)}^{\hat{D}_R(L)} = \frac{\overbrace{\left(\sum_n \hat{f}_n(0)\hat{b}_n(L)\right)}^{\hat{D}_R^{(in)}}\, h\, e^{\frac{1}{2}i\gamma\, k\, \Delta P\, L}\, i - \overbrace{\left(\sum_n \hat{b}_n(L)\hat{b}_n^*(L)\right)}^{=1}\, \hat{D}_R(L)\, \sin(Th)\, (v+w)}{h\, \cos(Th)\, i - v\, \sin(Th)}$$

that can be recast in the following implicit equation for $\hat{D}_R(L)$:

$$\hat{D}_R(L) = \frac{\hat{D}_R^{(in)}\, h\, e^{\frac{1}{2}i\gamma\, k\, \Delta P\, L}\, i - \hat{D}_R(L)\, \sin(Th)\, w}{h\, \cos(Th)\, i} \tag{S6}$$

Where $\hat{D}_R^{(in)} = \sum_n \hat{f}_n(0)\hat{b}_n(L)$ represents the correlation among the input FS and the input BCB, which is fixed by the boundary conditions. Note that $\hat{D}_R^{(in)}$ and $D_R^{(in)} = \sum_n f_n(0)\, b_n(L)/\left(P_f\, P_b\right)^{1/2}$ are identical up to a phase term and share therefore the same magnitude. Eq.(S6) can be solved numerically for $\hat{D}_R(L)$ in the complex domain. It is however useful to derive an equation for the magnitude $|\hat{D}_R(L)| \equiv |D_R(L)|$. We multiply the left and right hand side of Eq. (S6) by $h\, cos(Th)\, i$ and then recast Eq. (S6) as:

$$\hat{D}_R(L)(h\, \cos(Th)\, i + \sin(T\, h)\, w) = \hat{D}_R^{(in)}\, h\, e^{\frac{1}{2}i\gamma\, k\, \Delta P\, L}\, i \tag{S7}$$

T, *h* and *w* are real-valued and we can therefore readily compute the magnitude of the left and right side of Eq.(S7). Finally, we rewrite $|\hat{D}_R(L)|^2 = h^2 - v^2$ and we obtain the relation for mode rejection Eq.(3) (reported here below as Eq. (S8) for convenience):

$$\sin^2(Th) = \frac{\left(h^2 - |D_R^{(in)}|^2 - v^2\right)\cdot h^2}{(h^2 - v^2)(h^2 - w^2)} \tag{S8}$$

It is worth noting that the steps to derive the relation Eq.(4) for mode attraction from Eqs.(2) are basically the same. The starting point is again the stationary problem with $\gamma_{nn} = \gamma$ and $\gamma_{nm} = (1/2)\gamma$ ($n \neq m$). Then, by applying the transformation Eqs.(S2), we obtain:

$$\begin{aligned} \partial_z \hat{f}_n &= \; i\gamma k P_b \hat{D}_A \hat{b}_n \\ \partial_z \hat{b}_n &= \; -i\gamma k P_f \hat{D}_A^* \hat{f}_n \end{aligned} \tag{S9}$$

Where now the correlation coefficient reads $\hat{D}_A = \sum_n \hat{f}_n \hat{b}_n^*$ (as anticipated, the subscript *A* stands for attraction) and:

$$\partial_z \hat{D}_A = i\gamma k \hat{D}_A\, P_{tot} \tag{S10}$$

It should be noted that Eqs.(S9) and (S10) are formally identical to Eqs.(S3) and (S4) after replacing $\hat{b}_n^* \rightarrow \hat{b}_n$, $P_f \rightarrow -P_f$, $\hat{D}_R \rightarrow \hat{D}_A$. This allows readily obtaining the relation for mode attraction, Eq.(4) (reported here below as Eq. (S11) for convenience):

$$\sin^2(Th') = \frac{\left(h'^2 + |D_A^{(in)}|^2 - w^2\right) \cdot h'^2}{\left(h'^2 - v^2\right)\left(h'^2 - w^2\right)} \quad \text{(S11)}$$

In order to obtain an estimate for $|\widehat{D}_R(L)|$, it proves useful to represent graphically Eq.(S8). For the sake of simplicity, here we analyze the case where forward and backward powers are identical ($\Delta P = 0$) that is reported in Fig.(S1), for which $h = |\widehat{D}_R(L)| \equiv |D_R(L)|$, v=0, w=1 and consequently the right-hand-side of Eq.(S8) reads $(h^2 - |D_R^{(in)}|^2)/(h^2 - 1)$. The solutions of Eqs.(S8) are the points at the intersection between the left-hand-side and the right-hand-side. When the coefficient T is large enough, then multiple solutions could be found, namely h1, h2 and h3 in the example of Fig.S1. Each solution has a complex counterpart that solves Eq.(S6) in the complex domain.
Despite the possible existence of multiple solutions, however when we simulate the full spatio-temporal dynamics Eqs.(1) we observe that the system systematically relaxes towards the lower solution , that is h1 in Fig.S1. We conjecture that this may depend on the initial boundary condition for Eqs.(1) that defines the field inside the fibre at time t=0, namely $f_n(z,t=0)$ and $b_n(z,t=0)$. This topic is still under investigation.

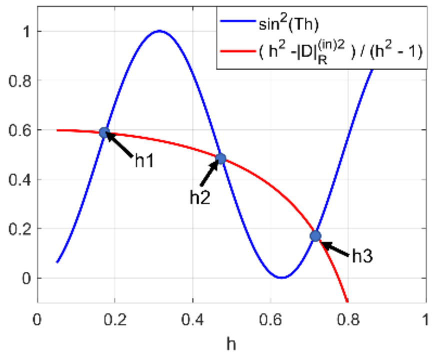

Fig.S1: Graphical representation of left-hand-side (blue line) and right-hand-side (red line) of Eq.(S8) when $\Delta P = 0$

In a highly nonlinear regime (T>>1) the solution h1 ~ 0 (see Fig.S1), and consequently the right-hand-side $(\mathrm{h1}^2 - |D_R^{(in)}|^2)/(\mathrm{h1}^2 - 1^2) \sim |D_R^{(in)}|^2$. Accordingly, Eq.(S8) becomes $\sin^2(Th) \sim |D_R^{(in)}|^2$, from which we obtain $h \sim asin(|D_R^{(in)}|)/T$ and therefore (since $h = |\widehat{D}_R(L)|$) the estimate $|D_R(L)| \sim asin(|D_R^{(in)}|)/T$ reported in the manuscript (Methods). With a similar procedure, from Eq.(S11) we obtain the estimate in the case of mode attraction:

$$|D_A(L)| \sim \left[1 - acos\left(|D_A^{(in)}|\right)^2 / T^2\right]^{1/2}.$$

Note 2. Universality of mode rejection and mode attraction phenomena

In this section we illustrate a few examples confirming the validity of the theoretical framework set out in section Note 1. We start from the case of mode rejection in the ideal case $\gamma_{nn} = \gamma$ and $\gamma_{nm} = (1/2)\gamma$, for which the estimate $|D_R(L)| \sim asin(|D_R^{(in)}|)/T$ holds true. Here we compare this estimate against the results obtained from the numerical solution of Eqs.(1). We consider the case of a L=1m long fibre supporting 4 modes and with γ=1/W/km. FS and BCB are co-polarised and have identical power (P_f=P_b=5 kW). While the input BCB is coupled to mode-1 only, the modes of the input FS have a relative power $|f_1(0)|^2/P_f$ =0.40, $|f_2(0)|^2/P_f$ = 0.27, $|f_3(0)|^2/P_f$ =0.12 and $|f_4(0)|^2/P_f$=0.21 , respectively. Since the BCB is coupled to mode-1 only, then $|D_R{}^{(in)}|^2 =$

$|f_1(0)|^2/P_f$ and $|D_R(L)|^2 = |f_1(L)|^2/P_f$. Therefore, in this specific case $\left|D_R{}^{(in)}\right|^2$ and $|D_R(L)|^2$ indicate the relative power coupled to mode-1 in the input and output FS, respectively. The first is fixed by the boundary conditions, that is $\left|D_R{}^{(in)}\right|^2 = |f_1(0)|^2/P_f = 0.40$. The latter can be evaluated via our theoretical estimate, that is $|D_R(L)|^2 = |f_1(L)|^2/P_f \sim asin(\,|D_R^{(in)}|\,)^2/T^2 = 0.019$.
Fig.S2 reports the results obtained from the numerical solution of Eqs.(1). Note that, differently from Eqs.(S1) that describes the stationary problem, Eqs.(1) account for the full space-time dynamics and then allow investigating the temporal evolution of the FS. When the BCB is off, there is no power exchange among the modes, therefore the mode power distribution of the output FS mirrors the input one. However, when the BCB is on, after a short transient time the output FS relaxes towards a stationary state. As predicted, rejection of mode-1 takes place in the output FS: the relative power of mode-1 goes from 0.40 when the BCB is off (Fig.S2a) down to ~0.018 when the BCB is on (Fig.S2b), in excellent agreement with our estimation of 0.019.

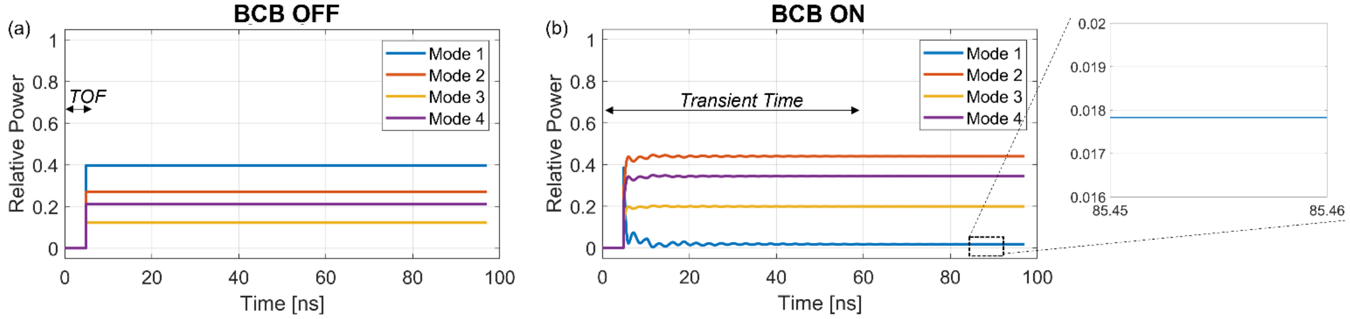

*Fig.S2: Temporal evolution of the output FS relative power $|f_n(t,L)|^2/P_f$ of mode-n (n={1,2,3,4}) from the simulation of Eqs.(1). When the BCB is off (**a**), the mode content of the output FS is unchanged with respect to the input one. The time delay TOF indicates the time-of-flight. When the BCB is on (**b**), then after a short transient time the system relaxes towards a stationary state. The input BCB is coupled to mode-1, therefore the output FS rejects mode-1, whose output relative power $|f_1(t,L)|^2/P_f$ is as low as ~0.018 when the stationary state is achieved.*

One may perform a similar analysis in the case of mode attraction. We simulate Eqs.(2) (again in the ideal case $\gamma_{nn} = \gamma$ and $\gamma_{nm} = (1/2)\gamma$) and we consider the same input conditions and parameters used in the previous example. In analogy with the previous case, $\left|D_A{}^{(in)}\right|^2 = |f_1(0)|^2/P_f$ and $|D_A(L)|^2 = |f_1(L)|^2/P_f$ describes the input and output relative power carried by the forward mode-1, respectively. The results are illustrated in Fig.S3. Now mode attraction takes place in the output FS. The relative power of mode-1 goes from 0.40 when the BCB is off (Fig.S3a) up to ~0.97 when the BCB is on (Fig.S2b),once again in excellent agreement with our estimate ($|D_A(L)|^2$=$|f_1(L)|^2/P_f \sim 1 - acos\left(|D_A^{(in)}|\right)^2/T^2$ =0.968.

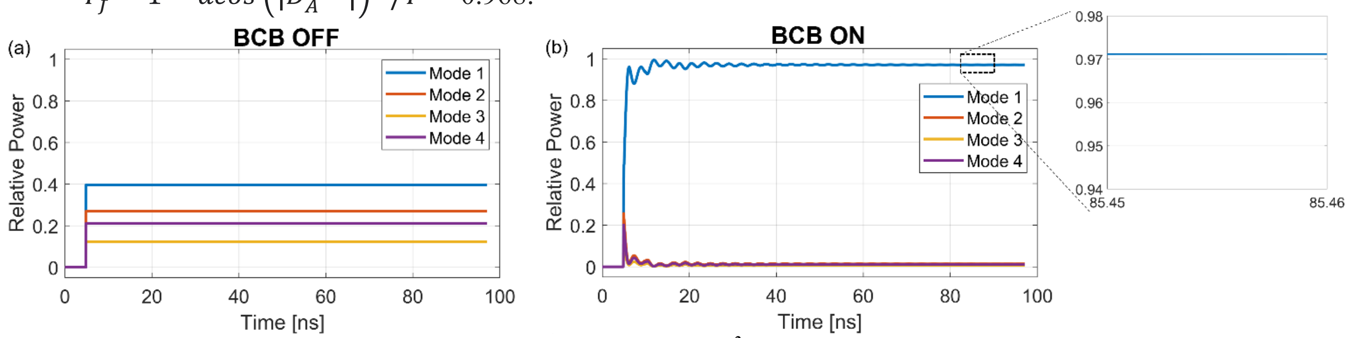

*Fig.S3: Temporal evolution of the output FS relative power $|f_n(t,L)|^2/P_f$ of mode-n (n={1,2,3,4}) from the simulation of Eqs.(2). When the BCB is off (**a**), the mode content of the output FS is unchanged with respect to the input one. Mode attraction towards mode-1 takes place when the BCB is on (**b**): the output relative power $|f_1(t,L)|^2/P_f$ grows up to ~0.97.*

A striking feature of Eqs.(1,2) is that they exhibit the same rejection and attraction dynamics even in the general case where the Kerr coefficients γ_{nm} are arbitrary. Indeed, we still observe a relaxation

towards a stationary state and again we identify the nonlinear interaction among forward and signal modes as the underpinning driving force leading to rejection or attraction.
To prove this, we have run 500 simulations of Eqs.(1) with 4 modes and by using an arbitrary set of Kerr-coefficients (see the caption of Fig.S4 for details). In each simulation, the fiber length is 1 m, the total FS and BCB power is 5kW and the BCB is coupled to mode-1. However, the relative power and phase of the input FS modes is random, which results in dispersed power distribution functions as reported in Fig.S4, top row. The bottom row of Fig.S4 shows instead the corresponding output power distribution function, from which we clearly observe an effective rejection of mode-1. Indeed, in all the 500 instances the power of the output FS coupled to mode-1 is lower than 10% (and <5% in 430 out of 500 instances). It is worth noting that these outcomes hold true even in the case where the input FS fluctuates in time, provided that the time scale of the fluctuations is longer than the characteristic response time of the system [1], which here can be roughly approximated with $c/(\gamma_{avg}\cdot P_f)$, γ_{avg} being the average Kerr coefficient.
We have performed the same numerical analysis in the case of Eqs.(2), and as shown in Fig.S5 we observe now a clear attraction towards mode-1. Indeed, in all the 500 instances most of the output FS power is carried by mode-1, and consequently the relative power of the others mode is almost null (seeFig.S5, bottom row). These results seem therefore to indicate that mode rejection and attraction are universal features of multimode nonlinear systems ruled by Eqs.(1) and (2), respectively.

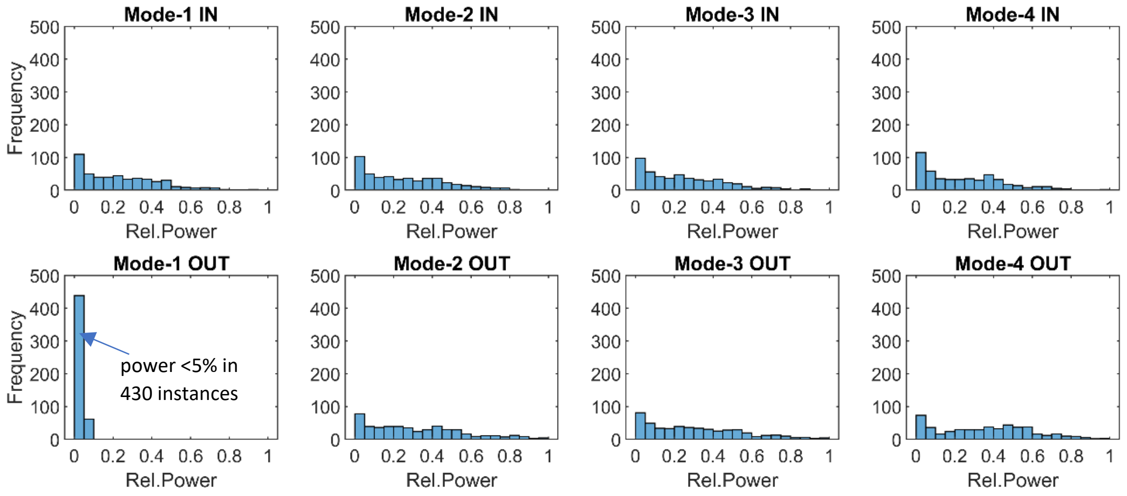

Fig.S4: Distribution function of the input (top row) relative powers $|f_n(t,0)|^2/P_f$ and of the output (bottom row) relative powers $|f_n(t,L)|^2/P_f$ of the FS when simulating 500 instances of Eqs.(1). The output relative powers reported in the bottom row are those achieved after the transient time (stationary value). The following set of Kerr coefficients is used in each simulation: γ_{11}=1/W/km; γ_{22}=1/W/km; γ_{33}=1.1/W/km; γ_{44}=1.2/W/km; γ_{12}= γ_{21}=0.7/W/km; γ_{13}= γ_{31}=0.6/W/km; γ_{14}= γ_{41}=0.5/W/km; γ_{23}= γ_{32}=0.7/W/km; γ_{24}= γ_{42}=1/W/km; γ_{34}= γ_{43}=0.9/W/km.

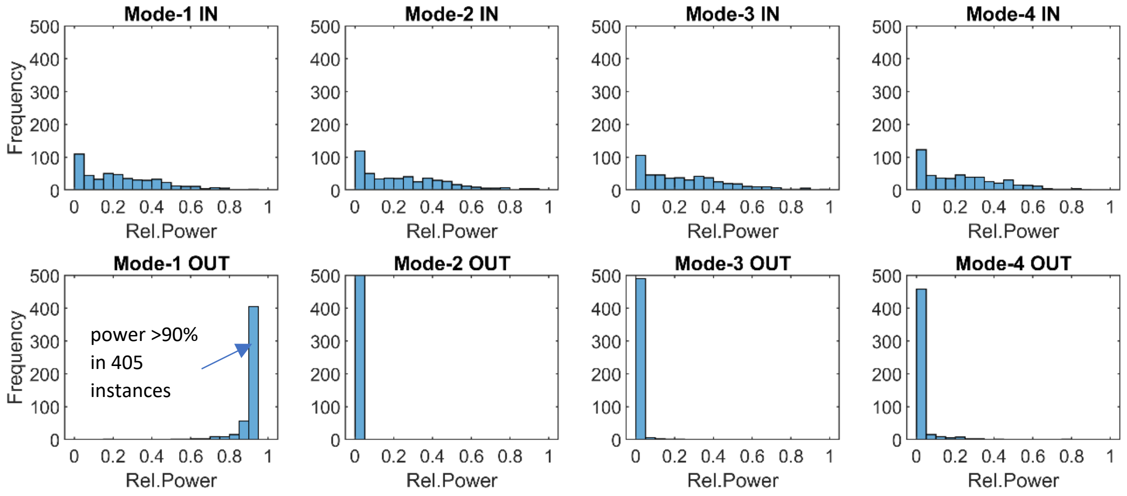

Fig.S5: Same as Fig.S4 but for simulation of Eqs.(2).

Note 3. Fibre characterization and experimental parameters

Tables S1 and S2 show the microscope measurements of the fibre diameter, core size and pitch for the dual-core-fibre (DCF) and three-core-fibre (TCF) fabricated in our cleanrooms. The measurements are taken over two fibre sections, at the start and then at the end of the pulled fibre, whose total length is about 1 km. Note that in the experiments we use however short fibre segments (1m for the DCF, 40 cm for the TCF). While ideally the cores size Dc1, Dc2 and Dc3 should be identical (5 um), in practice due to fabrication errors the difference is up to ~4% in the same section. Similarly, there is a ~2% variation from the core-to-core pitch P12 to P13 in the TCF.

Table S1: Microscope measurements of the DCF

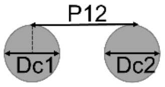

DCF	OD(μm)	Dc1(μm)	Dc2(μm)	P12(μm)
Start	141.9	4.92	5.06	9.58
End	140.7	4.86	5.09	9.31

OD: outer fibre diameter, Dc1,Dc2: diameter of core1,core2; P12: distance between the centres of core1 and 2.

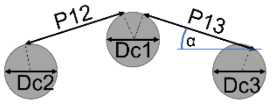

Table S2: Microscope measurements of the TCF

TCF	OD(μm)	Dc1(μm)	Dc2(μm)	Dc3(μm)	P12(μm)	P13(μm)
Start	137.9	4.43	4.51	4.41	9.22	9.34
End	137.9	5.07	5.11	4.98	9.16	9.36

OD: outer fibre diameter, Dc1,Dc2,Dc3: diameter of core1,core2,core3; P12: distance between the centres of core 1 and 2.; P13: distance between the centres of core 1 and 3; α= 30 deg.

The microscope measurements reported above, along with a standard measurement of the refractive index profile, are then used to model the DCF and the TCF with a finite-element-method software (Comsol Multyphisics 5.6), and then to compute the spatial mode profiles (reported in Fig.2b), the group velocity and the dispersion of each spatial mode, as well as the effective areas from which we derive the nonlinear Kerr coefficients, reported in Table S3 and S4. Note that both the DCF and the TCF are polarization-maintaining and modes are linearly polarized along the horizontal or vertical direction. This is confirmed by our experiments, where we find an extinction-ratio >10 dB. The data reported in Tables S3 and S4 are almost independent of the mode polarization.

Table S3: estimation of the DCF parameters

γ_{11}(kW^{-1}m^{-1})	γ_{22}(kW^{-1}m^{-1})	γ_{12}(kW^{-1}m^{-1})	
3	3.12	3.06	
β_1^{SMe}(ps/mm)	β_1^{SMo}(ps/mm)	β_2^{SMe}(ps^2/km)	β_2^{SMo}(ps^2/km)
4.906	4.907	23.577	20.609

$\gamma_{11(22)}$: intramodal nonlinear Kerr coefficient of the $SM_{e(o)}$ mode, γ_{12}: intermodal Kerr coefficient between the SM_e and the SM_o mode; β_1, β_2: inverse group velocity, and group velocity dispersion coefficient of the modes.

Table S4: estimation of the TCF parameters

γ_{11}(kW^{-1}m^{-1})	γ_{22}(kW^{-1}m^{-1})	γ_{33}(kW^{-1}m^{-1})	γ_{12}(kW^{-1}m^{-1})	γ_{13}(kW^{-1}m^{-1})	γ_{23}(kW^{-1}m^{-1})
2.3	3.17	2.46	1.56	2.37	1.61
β_1^{SM1}(ps/mm)	β_1^{SM2}(ps/mm)	β_1^{SM3}(ps/mm)			
4.906	4.907	4.907			
β_2^{SM1}(ps^2/km)	β_2^{SM2}(ps^2/km)	β_2^{SM3}(ps^2/km)			
24.425	22.140	19.554			

$\gamma_{11(22,33)}$: intramodal nonlinear Kerr coefficient of the $SM_{1(2,3)}$ mode, γ_{mn}: intermodal Kerr coefficient between the SM_m and the SM_n mode; β_1, β_2: inverse group velocity, and group velocity dispersion coefficient of the modes.

For each one of the experiments illustrated in Fig.3 (DCF) and Fig.5 (TCF), we report in Table S5 the corresponding peak power of the input FS and input BCB, along with their mode decomposition. Note

that the power P_f of the input FS is fixed, whereas the power P_b of the BCB is gradually increased from 0 to its maximum value.
The values of peak power have been chosen to achieve a high nonlinear regime, which is necessary condition to observe the mode rejection dynamics. As reported in the manuscript (section Methods) the parameter $T=(T_f T_b)^{1/2} = L\gamma(P_f P_b)^{1/2}$ represents the overall system nonlinearity. As a rule of thumb, our simulations indicate that we need $T>2$ to observe effective mode rejection. In the fibres under test, where the Kerr coefficients are generally different each other, γ is replaced by their arithmetic average γ_{avg}. Moreover, because we use pulsed beams, the fibre length L is replaced by the actual interaction length $L_{in}=2\cdot t_0\cdot c$ (c=speed of light in the fibre, t_0=pulse width, 0.5 ns). In conclusion, the parameter $T_{eff} = L_{in}\gamma_{avg}(P_f P_b)^{1/2}$, reported in the last row of Table S5, is used to evaluate the system nonlinearity. The peak powers P_f and P_b are properly chosen so as to meet the condition $T_{eff}>2$.
The mode decomposition provides an estimation of both the relative power and relative phase for each mode. In the case of the DCF (Fig.3a,b,c) the relative phase reported in Table S5 is the one between SM_e and SM_o. In the case of the TCF (Fig.5a,b,c) the first value indicates the relative phase between SM_1 and SM_2, the second value between SM_1 and SM_3.

Table S5: Experimental parameters

		Fig. 3a	Fig. 3b	Fig. 3c	Fig. 5a	Fig. 5b	Fig. 5c
Input FS	Fixed Peak power $\mathbf{P_f}$ (kW)	3.75	6.5	6.2	4.23	4.23	5.33
	Mode Rel.Power(%)	SMe=58<br>SMo=42	SMe=59<br>SMo=41	SMe=28<br>SMo=72	SM1=40<br>SM2=18<br>SM3=42	SM1=36<br>SM2=17<br>SM3=47	SM1=12<br>SM2=37<br>SM3=51
	Mode Rel. phase (rad)	3.1	3	2.9	2.3, 0.1	2.3, 0.1	6, 2.1
Input BCB	Mode Rel.Power(%)	SMe=0<br>SMo=100	SMe=100<br>SMo=0	SMe=100<br>SMo=0	SM1=100<br>SM2=0<br>SM3=0	SM1=0<br>SM2=100<br>SM3=0	SM1=0<br>SM2=0<br>SM3=100
	Max Peak power $\mathbf{P_b}$ (kW)	5.08	5.58	5.58	6.18	4.48	5.33
	T_{eff} (max)	2.67	3.69	3.60	3.13	2.66	3.26

When running our numerical simulations of Eqs.(1), which are reported in Fig.3 and Fig.5 along with the experimental results, we have used the fibre and experimental parameters reported in Tables S3-S5. The temporal pulses are modelled with a super-gaussian shape of 6th order. The pulse width (500 ps in the experiments) is the only free-parameter in our simulations. It is adjusted between 500ps and 700ps to get the good quantitative agreement with experiments illustrated in Figs.3 and Fig.5.
Note that in the case of the PM-6MF, we were unable to fit quantitatively the experimental results via numerical simulations. Indeed, our simulations indicates that when the number of modes becomes significant (typically >4), then the specific dynamic of each individual mode is extremely sensitive to small variations of the input conditions and fibre parameters. This however does not change the fundamental outcome, namely mode rejection of the BCB mode is always achieved. Finally, note that the estimated beat-length among the pairs of modes (LP_{11a}, LP_{11b}) and (LP_{21a}, LP_{21b}) is < 1mm, therefore much shorter than the fibre employed in the experiments. This makes the fibre to support in effect 6 distinct modes rather than just 4. As in the case of the DCF and TCF, the PM-6MF is polarization-maintaining, with a polarization extinction-ratio >18 dB. In Fig.S6 we represent the birefringent axes of the DCF, TCF and PM-6MF.

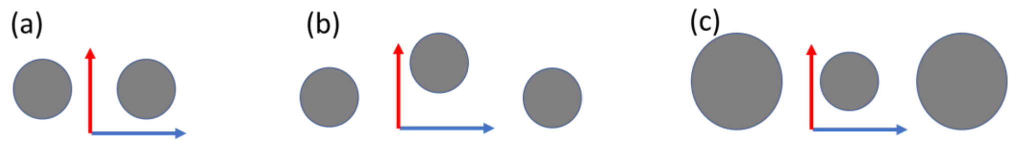

*Fig.S6: Birefringence axes (horizontal axis in blue, vertical axis in red) for the DCF (**a**), TCF (**b**) and PM-6MF (**c**)*

Note 4. Movies

We have recorded some Supplementary movies illustrating the rejection dynamics in the fibres under tests. Supplementary movies 1-3 report the results illustrated in Fig.3a, 3b and 3c, respectively. Supplementary movies 4-6 report the results illustrated in Fig.5a, 5b and 5c, respectively. Supplementary movies 7-9 report the results illustrated in Fig.6a, 6b and 6c, respectively. In each movie, the input FS is launched with a fixed power (see Note 3 for experimental parameters). On the contrary, the input BCB power is gradually increased from 0 to its maximum value. For a given BCB power, each movie frame reports the following information (see Fig.S7):

(1) the current BCB power;

(2) the corresponding relative power of the rejected mode in the output FS (obtained from mode decomposition);

(3) the measured far-field intensity profile of the output FS;

(4) the reconstructed far-field intensity profile obtained when using the mode weights and phase from mode decomposition;

(5) the 2D spatial correlation coefficient (Corr) between the measured and the reconstructed far-field [2]. It should be noted that Corr is typically >98%, which attests the accuracy of our mode decomposition algorithm.

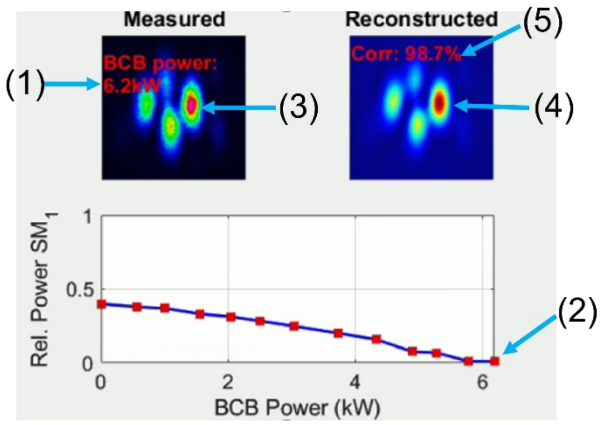

Fig. S7. Example of video frame from Figure5a.mov and related information

Note 5. Schematic summary

Here below a schematic summary that provides a conceptual map of the main results discussed in this paper.

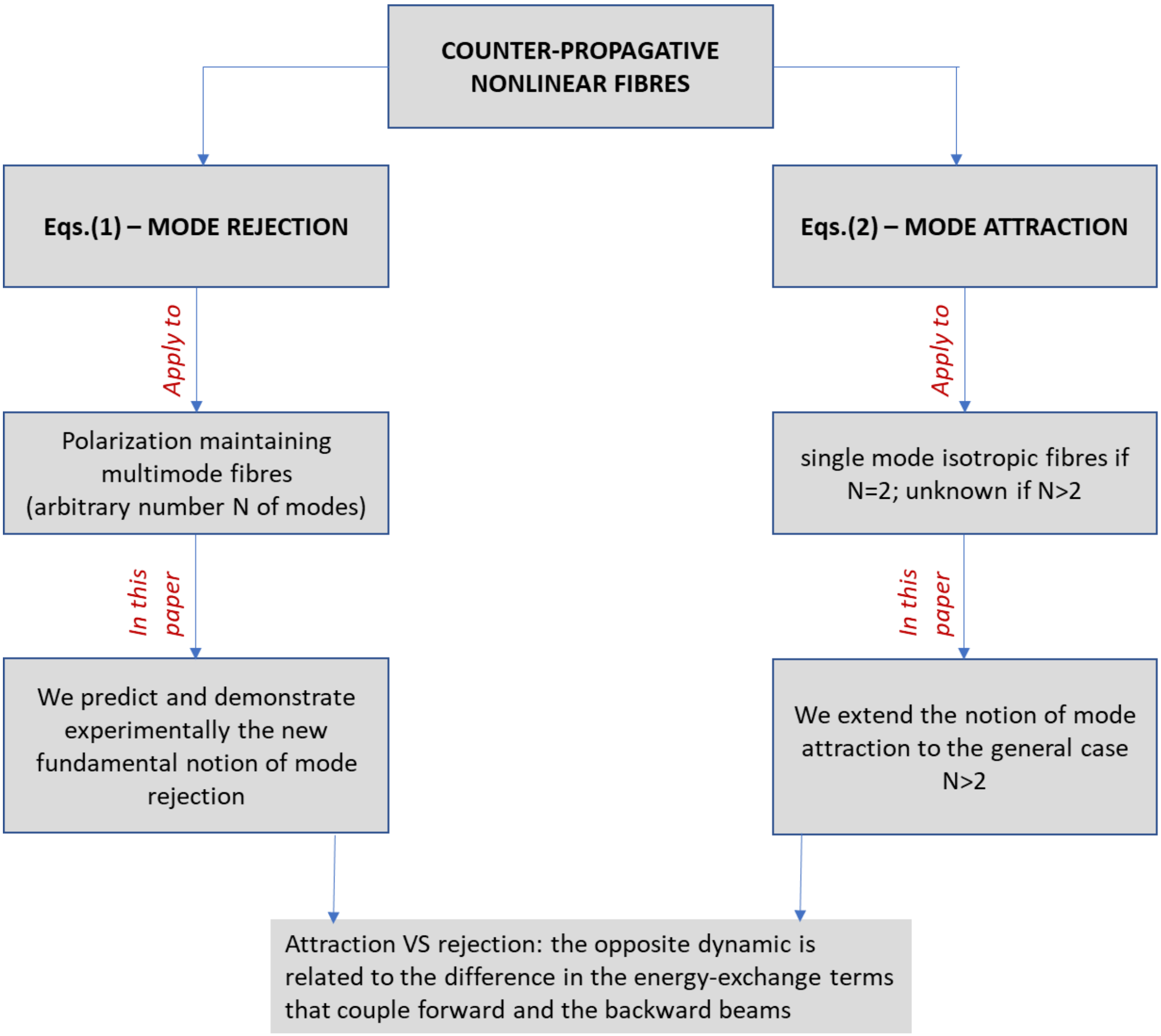

REFERENCES

1. V.V. Kozlov, J.Fatome, P. Morin, S. Pitois, G. Millot, and S. Wabnitz Nonlinear repolarization dynamics in optical fibers: transient polarization attraction. *J.Opt.Soc.Am. B* **28**, 1782-1791 (2011)

2. R. Bruning, P. Gelszinnis, C. Schulze, D. Flamm, and M. Duparre, Comparative analysis of numerical methods for the mode analysis of laser beams, *Appl. Opt.* **52**, 7769-7777 (2013)
