# Supplementary material for: Mode attraction, rejection and control in nonlinear multimode optics
Source: Nat Commun. 2023 Nov 24;14:7704. doi: 10.1038/s41467-023-42869-0 (PMC10673906; doi:10.1038/s41467-023-42869-0)
Supplement: Supplementary file 3 — Description of Additional Supplementary Files [file 41467_2023_42869_MOESM3_ESM.docx]

**Description of Additional Supplementary Files**

**Supplementary Movie 1:** Measured and reconstructed far-field of the output FS as a function of the BCB power. Fibre parameters and mode decomposition are those related to figure 3a in the manuscript. See Supplementary information note 4 for details.

**Supplementary Movie 2:** Measured and reconstructed far-field of the output FS as a function of the BCB power. Fibre parameters and mode decomposition are those related to figure 3b in the manuscript. See Supplementary information note 4 for details.

**Supplementary Movie 3:** Measured and reconstructed far-field of the output FS as a function of the BCB power. Fibre parameters and mode decomposition are those related to figure 3c in the manuscript. See Supplementary information note 4 for details.

**Supplementary Movie 4:** Measured and reconstructed far-field of the output FS as a function of the BCB power. Fibre parameters and mode decomposition are those related to figure 5a in the manuscript. See Supplementary information note 4 for details.

**Supplementary Movie 5:** Measured and reconstructed far-field of the output FS as a function of the BCB power. Fibre parameters and mode decomposition are those related to figure 5b in the manuscript. See Supplementary information note 4 for details.

**Supplementary Movie 6:** Measured and reconstructed far-field of the output FS as a function of the BCB power. Fibre parameters and mode decomposition are those related to figure 5c in the manuscript. See Supplementary information note 4 for details.

**Supplementary Movie 7:** Measured and reconstructed far-field of the output FS as a function of the BCB power. Fibre parameters and mode decomposition are those related to figure 6a in the manuscript. See Supplementary information note 4 for details.

**Supplementary Movie 8:** Measured and reconstructed far-field of the output FS as a function of the BCB power. Fibre parameters and mode decomposition are those related to figure 6b in the manuscript. See Supplementary information note 4 for details.

**Supplementary Movie 9:** Measured and reconstructed far-field of the output FS as a function of the BCB power. Fibre parameters and mode decomposition are those related to figure 6c in the manuscript. See Supplementary information note 4 for details
